# Transcriptional and Post-Transcriptional Anticholestatic Mechanisms of Obeticholic Acid in Lipopolysaccharide-Induced Cholestasis

**DOI:** 10.3390/pharmaceutics17111393

**Published:** 2025-10-28

**Authors:** María Valeria Razori, Geraldine L. Hillotte, Pamela L. Martín, Ismael R. Barosso, Cecilia L. Basiglio, María Laura Ruiz, Marcelo G. Roma

**Affiliations:** Institute of Experimental Physiology (IFISE), Department of Physiological Sciences, Faculty of Biochemical and Pharmaceutical Sciences, National University of Rosario, Rosario 2000, Argentinahillote@ifise-conicet.gov.ar (G.L.H.);

**Keywords:** obeticholic acid, hepatocellular transporters, multidrug resistance-associated protein 2, bile salts, Bsep, lipopolysaccharide-induced cholestasis

## Abstract

**Background/Objectives:** Sepsis-induced cholestasis is caused by the release of inflammatory cytokines from lipopolysaccharide (LPS), a component of Gram-negative bacteria. No established therapy exists for this condition. We ascertained the anticholestatic potential of obeticholic acid (OCA), a potent FXR agonist, in a rat model of LPS-induced cholestasis. **Methods:** Male Wistar rats were randomized into Control, OCA (20 mg/kg/day, i.p., 6 days), LPS (total dose of 6.5 mg/kg, i.p., in the last 2 days, respectively), and OCA + LPS groups. Then, we assessed the serum cholestasis marker, alkaline phosphatase (ALP), and taurocholate-stimulated bile salt output. mRNA/protein levels of the main apical and sinusoidal uptake and efflux carriers were assessed by either or both RT-qPCR and Western blot. Bsep and Mrp2 localization was assessed by immunohistochemistry followed by confocal microscopy and image analysis. Inflammatory cytokines were measured in serum by ELISA. **Results:** OCA significantly attenuates inflammatory cytokine release and normalizes serum ALP in LPS-treated rats. OCA also increased the biliary output of the Bsep substrate, taurocholate, and partially improved total Bsep at both mRNA and protein levels. Furthermore, OCA fully normalizes Bsep in the canalicular plasma membrane fraction, suggesting improved membrane localization, a finding further confirmed by confocal microscopy. OCA sustained the beneficial downregulation of uptake transporters Ntcp and Oatp2 or the upregulation of the efflux pump Mrp3, both of which serve to minimize hepatocellular bile-salt accumulation. **Conclusions:** OCA prevents bile-salt accumulation in LPS-induced cholestasis by enhancing Bsep expression and localization, and by mitigating inflammation. This makes OCA a promising therapeutic candidate for sepsis-induced cholestasis.

## 1. Introduction

Cholestasis is a frequent and often severe clinical condition associated with numerous liver diseases [1]. Sepsis-induced cholestasis is a form of functional cholestasis characterized by impaired bile secretion [2,3]. This condition has been linked to the cholestatic effects of lipopolysaccharides (LPS), components of the bacterial cell wall (endotoxins), and the subsequent release of proinflammatory cytokines, primarily produced by LPS-stimulated Kupffer cells [3]. LPS administration to rodents has been widely used as an experimental model for this type of cholestasis [4].

LPS-induced cholestasis results from transcriptional and post-transcriptional alterations in the expression of key solute transporters involved in bile formation, primarily those responsible for the transport of bile salts (BSs) and glutathione (GSH) [2]. The expressions of bile salt export pump (Bsep/BSEP; ABCB11), the main canalicular BS transporter, and multidrug resistance-associated protein 2 (Mrp2/Abcc2), which mediates the canalicular excretion of bilirubin, GSH, and glucuronidated/sulfated BSs [1], are impaired in LPS-induced cholestasis in rodents, at least in part, through transcriptional mechanisms [5]. Similarly, at the basolateral pole, expression of the main basolateral BS uptake transporter, Na^+^/taurocholate cotransporting polypeptide (Ntcp/NTCP; Slc10a1/SLC10A1), as well as certain isoforms of organic anion transport protein (Oatp/OATP; slc21a/SLC21A), which are also involved in BS uptake, is decreased at the transcriptional level, to limit BS uptake and further harmful intracellular accumulation of BSs [5]. In addition, at the basolateral membrane, multidrug resistance-associated protein 3 (Mrp3/Abcc3) is adaptively upregulated in LPS-induced cholestasis [6], to extrude accumulated monovalent BSs (glyco- and tauro-conjugates) and divalent BSs (e.g., sulfo- and glucurono-conjugates) [7]; this reduces the concentration of potentially harmful BSs in hepatocytes by providing an alternative route of excretion via urine.

The transcriptional changes in hepatocellular transporters in inflammatory cholestasis are mainly due to repressed expression of nuclear receptors with a transactivating effect on the synthesis of the affected carriers [8]. One key factor is the reduced expression of retinoid X receptor (RXRα), as this nuclear receptor heterodimerizes with many other nuclear receptors to promote nucleus-to-cytoplasm transfer [9,10]. This alteration is reinforced by the downregulation of the expression of other nuclear receptors that interact with RXRα to regulate hepatocellular secretory function. For example, farnesoid X receptor (FXR), the main nuclear receptor involved in BS homeostasis regulation, is downregulated by proinflammatory cytokines via inhibition of the transactivating effect of hepatic nuclear factor 1α (HNF1α) on the FXR gene promoter [11]. This establishes a vicious cycle that reinforces the inflammatory effects of LPS, since FXR downregulation impairs FXR-induced repression of nuclear factor kappa B (NF-κB), a transcription factor that is activated by LPS to trigger cytokine release [12].

In addition to these transcriptional mechanisms of cholestasis, LPS is associated with short-term, post-translational changes in the normal localization of canalicular transporters within their plasma membrane domain. For example, both Bsep and Mrp2 undergo rapid endocytic internalization and concomitant accelerated degradation when the rat liver is exposed to LPS [13]; this phenomenon could explain the selective post-transcriptional downregulation of these transporters in LPS-induced cholestasis in humans [14].

Obeticholic acid (OCA, also known as INT-747) is a semi-synthetic BS (6α-ethyl chenodeoxycholic acid) and a potent, selective agonist of FXR. Its anticholestatic properties have been demonstrated in various rat models of cholestasis, including those induced by α-naphthylisothiocyanate [15], estrogens [16], and lithocholic acid [17]. OCA exhibits approximately 100-fold greater FXR agonistic activity than chenodeoxycholic acid, the primary natural FXR agonist in humans [18].

The pharmacological administration of high-affinity FXR ligands can more efficiently promote the transcriptional changes induced by FXR, with additional therapeutic benefits [17]. OCA has been approved for the treatment of primary biliary cholangitis, a chronic cholestatic pathology where inflammation may play a central role [19]. Stimulatory effects of OCA on BS output, with the consequent reduction in hepatocellular BS exposure, have been suggested in this condition [20]. The transcriptional, FXR-mediated effects of OCA could explain many of its anticholestatic effects. FXR target genes include Bsep and several basolateral extrusion transporters, including Mrp3 [21]. In addition, OCA has anti-inflammatory properties likely associated with the capability of FXR to counteract the synthesis of proinflammatory cytokines by activated hepatic stellate cells [22], which can also play a crucial role in its anticholestatic effects in inflammatory cholestasis. OCA has been shown to mitigate the elevation of inflammatory cytokines in different experimental models of inflammatory cholestasis [15,23,24], including that induced by LPS [25].

Currently, there is no treatment for sepsis-induced cholestasis other than eradication of the underlying infection. However, cholestasis has extraordinary prognostic relevance for the course of sepsis. Hepatic dysfunction is a powerful, independent predictor of mortality [26], and cholestasis is an early event as part of this liver injury, as reflected by the often very early elevations of serum BSs in patients with severe sepsis [27]. Furthermore, BSs function as danger-associated molecular patterns (DAMPs) capable of activating the NLRP3 inflammasome in proinflammatory macrophages [28]. Therefore, the aim of this study is to explore the possibility of counteracting the changes in expression and localization of hepatocellular transporters in LPS-induced cholestasis by using OCA as an anticholestatic agent, based on the activation of transcriptional and post-transcriptional pathways mediated by this pharmacological compound.

## 2. Materials and Methods

### 2.1. Chemicals

LPS from *Salmonella typhimurium*, bromosulfophthalein (BSP), NADPH, and GSH reductase were purchased from Sigma-Aldrich (St. Louis, MO, USA). Sodium taurocholate (TC) was purchased from Santa Cruz Biotechnology (Dallas, TX, USA). All the other reagents were of the highest analytical grade available from commercial sources. OCA (≥96–98% pure) was kindly provided by Intercept Pharmaceuticals (San Diego, CA, USA).

### 2.2. Animals and Treatments

Adult, male Wistar rats (300–350 g) were used in all the studies. They were provided by the Center for Comparative Medicine at the Institute of Veterinary Sciences of Litoral (UNL-CONICET). Animals were kept on a standard diet with free access to water and saline, and exposed to a constant light cycle (12 h light/dark). Throughout the experiments, vital signs, including heart rate and respiratory rate, as well as pain sensitivity, were regularly monitored. Humane care was provided to all animals in compliance with the “Guide for the care and use of laboratory animals” (NIH, publication 25–28, revised in 1996). All animal studies were conducted with the approval of the Bioethics Committee for the Care and Use of Laboratory Animals of the Biochemical and Pharmaceutical Science School of the National University of Rosario (Res. 489/2015, 6 February 2020).

Rats were randomly assigned to the following four experimental groups:

- LPS group: Animals were administered with OCA vehicle (saline), at the daily dose of 4.5 mL/kg of b.w, i.p., for 6 days, and with 3 single, i.p., injections of LPS (dissolved in saline), according to the following schedule: 2 LPS doses of 2.5 mg/kg/day of b.w. at 8:00 a.m. of the last 2 days, and an additional LPS dose of 5 mg/kg of b.w. at 8:00 p.m. of the last day [29].
- OCA + LPS group: Animals were injected with OCA, i.p., at the dose of 20 mg/kg of b.w., dissolved in saline solution (pH = 7.4) for 6 days; this was the minimum period of time required for OCA to reproducibly improve expression of its model target gene, *Bsep*. Animals were also administered 3 doses of LPS, as described above.
- Control group: Animals were injected only with OCA and LPS vehicle (saline solution).
- OCA group: Animals were injected only with OCA for 6 days, as described above.

### 2.3. Surgical Procedures

Twelve hours after the final LPS dose, animals received ketamine (100 mg/kg) and xylazine (3 mg/kg) i.p. for anesthesia, and were, thus, maintained throughout the experiments. Normothermia (36.0–37.5 °C) was controlled with a rectal probe and maintained with a heating lamp. Then, the femoral vein and the common bile duct were cannulated with PE50 and PE10 polyethylene tubing, respectively (Intramedic, Clay Adams, Parsippany, NJ, USA), for i.v. administrations and bile-sample collections, respectively. Upon completion of the experiments, the animals were exsanguinated, and blood and liver samples were harvested for subsequent analyses.

### 2.4. Bile/Plasma Samples Collection and Analytical Assessments

Bile was collected over a 10 min period to assess basal bile flow (BF). BF was measured gravimetrically, based on the assumption that bile has a density of 1 g/mL. Basal bile was assayed for total BSs, using the Randox 5th Generation Bile Acids Assay^®^ (Crumlin, Northern Ireland, UK), and for total GSH, using the recycling method of Tietze [30], as modified by Griffith [31].

Plasma samples were analyzed for alkaline phosphatase (ALP; EC 3.1.3.1) to assess cholestatic liver injury (Wiener Lab, Rosario, Argentina). A blood sample was also collected from the tail vein 2 h after the second LPS injection to measure plasma levels of the inflammatory cytokines, IL 1-β and TNF-α, by using commercial kits (IL-1 beta Rat ELISA Kit^®^ and TNF alpha Rat ELISA Kit^®^, Invitrogen, Waltham, MA, USA, respectively).

### 2.5. Mrp2 and Bsep Transport Activity Studies

Bsep and Mrp2 transport functions were assessed by using sodium TC and BSP as model substrates, respectively. Due to its higher affinity for Bsep in comparison to other BSs, TC is regarded as one of the most reliable substrates for evaluating Bsep-mediated transport [32]. As for BSP, this cholephilic dye is exported into bile with high affinity by Mrp2 after GSH conjugation by glutathione *S*-transferases [33], and its Mrp2-mediated transfer, but not its conjugation, is the rate-limiting step of its plasma-to-bile transport [34].

TC (8 μmol/100 g b.w.) was injected i.v. [28]. Then, bile was collected every 2 min for 10 min; this time period allows for the complete elimination of the exogenously administered TC in control animals, according to previous results [35]. Total BSs were then measured in bile. TC excretion was estimated by attributing the entire increase in biliary BS output, relative to the basal period, to TC, as previously validated by our group [35].

BSP was administered in a single, i.v. dose of 3 mg/100 g b.w., as described previously [35]. Bile samples were then collected in 5 min intervals over the next 60 min, a time period sufficient to fully depurate the dye in control animals. BSP concentrations in bile were measured spectrophotometrically at 580 nm, following appropriate dilution with 0.1 N NaOH [35].

### 2.6. Real-Time PCR Analysis of Canalicular and Basolateral Hepatocellular Transporters

Liver tissue was processed with TRIzol^®^ reagent (Invitrogen, Waltham, MA, USA) to isolate total RNA, according to the manufacturer’s instructions. The purity, yield and integrity of RNA were evaluated. Then, the synthesis of first-strand cDNA was carried out using SuperScript III Reverse Transcriptase^®^ and random primers (Invitrogen, Waltham, MA, USA), according to the manufacturer’s suggested protocol. Nucleotide sequences of primer pairs and conditions for assessment of Mrp2 [36], Bsep [35], Mrp3 [36], and 18S [36] gene expressions were as previously described. Sequences of primer pairs and conditions for Mrp4, Oatp2, and Ntcp were designed to optimally detect the respective mRNAs (Table 1). Real-time PCR reactions were carried out in a Mx3000P System^®^ (Stratagene, La Jolla, CA, USA) with 5× HOT FIREPol^®^ EvaGreen^®^ qPCR Mix Plus (ROX) (Solis BioDyne, Tartu, Estonia), according to the manufacturer’s instructions. Results were normalized to the expression of 18S rRNA as the housekeeping gene, by using the 2−ΔΔCt method [37].

### 2.7. Transporter Protein Expression in Liver

Assessment of protein levels of Bsep, Mrp2, and Ntcp by Western blotting in total homogenate and, for Bsep and Mrp2, in a subcellular fraction enriched in canalicular membrane markers, was carried out as described previously [38]. Protein samples were separated by electrophoresis on polyacrylamide gels under denaturing conditions. Following electrophoresis, proteins were transferred onto PVDF membranes. The membranes were then blocked for at least 1 h in a buffer containing 1X PBS, 0.3% Tween, and 5 g of low-fat dry milk in 50 mL. After blocking, membranes were incubated overnight at 4 °C with the appropriate primary antibodies (polyclonal rabbit anti-rat Bsep antibody, 1:1000, Anti-SPGP pAb, Kamiya Biomedical Company (Seattle, WA, USA); monoclonal mouse anti-rat Mrp2 antibody, 1:1000, M2 III-6, Alexis Biochemicals (Carlsbad, CA, USA); polyclonal rabbit anti-rat Ntcp antibody, 1:1000, NTCP Polyclonal Antibody, Invitrogen (Waltham, MA, USA). The following day, membranes were washed and incubated for 1 h with horseradish peroxidase-conjugated secondary antibodies to detect immune complexes. Immunoreactive bands were visualized using an enhanced chemiluminescence reagent (ECL^®^, Amersham Pharmacia Biotech, Inc., Piscataway, NJ, USA). Membranes were subsequently re-probed with an anti-β-actin antibody (Sigma-Aldrich Corp., St. Louis, MO, USA) as a loading control. Densitometry was performed to quantify band intensities using the Gel Pro Analyzer software, version 6.3 (Media Cybernetics, Silver Spring, MD, USA).

### 2.8. Immunofluorescence Detection of Bsep and Mrp2

Liver tissue samples were immersed in isopentane at −70 °C, and then promptly removed and stored at −70 °C until further use. The tissues were subsequently sectioned into 5 μm slices using a cryostat, and then fixed and stained according to previously described protocols [33]. Immunolabeling was carried out by incubating the tissue sections overnight with a rabbit anti-rat Bsep (1:100; Kamiya Biomedical Co., Seattle, WA, USA) or a mouse anti-rat Mrp2 (1:100; [M2III-6]; Alexis Biochemicals, San Diego, CA, USA), followed by incubation with a Cy2-conjugated donkey anti-rabbit IgG or a Cy2-conjugated donkey anti-mouse IgG, respectively (1:100, 1 h; Jackson ImmunoResearch Laboratory, West Grove, PA, USA). To delimit the bile canaliculi, the tight junction-associated proteins, occludin and ZO-1, were used when simultaneous Bsep and Mrp2 immunostaining were carried out, respectively. Occludin was stained with a mouse anti-rat occludin (1:100, overnight; Invitrogen, Waltham, MA, USA) and ZO-1 with a rabbit anti-rat occluding (1:100, overnight; Invitrogen, Waltham, MA, USA), followed by incubation with a Cy3-conjugated donkey anti-mouse and a Cy3-conjugated donkey anti-rabbit (1:100, 1 h, Jackson ImmunoResearch Laboratory, West Grove, PA, USA), respectively. Confocal images were acquired using an LSM880 microscope (Carl Zeiss LLC, Thornwood, NY, USA). To ensure consistency in staining and imaging across all experimental groups, liver sections were prepared on the same day, co-mounted on a single glass slide, and processed simultaneously for staining and microscopy. The degree of Bsep and Mrp2 endocytic internalization was quantified on confocal images using ImageJ 1.34 m (National Institutes of Health, Bethesda, MD, USA), as described by us elsewhere [39]. For this purpose, the intensity profiles of the transporter-associated were measured along an 8 μm line perpendicular to the canalicular axis, spanning from −4 μm to +4 μm relative to the canalicular center. For each tissue section, data were collected from a minimum of 30 different canaliculi and used for statistical analysis.

### 2.9. Statistical Analysis

The minimal number of animals per group required to obtain valid statistical results was calculated with the Resource Equation Method [40]. Data are expressed as means ± SEM. Statistical analyses were performed using ANOVA or Student’s *t*-test, unless an alternative test was deemed more suitable. The variances of the densitometric fluorescence profiles corresponding to Bsep and Mrp2 localizations were compared using the Mann–Whitney U test [35]. Values of *p* < 0.05 were considered to be statistically significant.

## 3. Results

### 3.1. Serum Parameters of Inflammatory Cholestasis

As shown in Table 2, LPS significantly increased ALP plasmatic levels, a plasma membrane enzyme that is upregulated and released from the cell surface of hepatocytes into blood by the detergent BSs retained in cholestasis [41]. OCA pretreatment fully counteracted this increase, thus indicating mitigation of BS accumulation.

Plasma levels of IL-1β and TNF-α were increased in LPS-treated animals by 2 h after the second dose of LPS, and OCA pretreatment significantly reduced these increments in a partial and total manner, respectively (Table 2). This suggests that the beneficial effects of OCA could be due, at least in part, to attenuation of the inflammatory response induced by LPS.

### 3.2. Biliary Parameters of Cholestasis

LPS significantly reduced BF, and OCA coadministration did not attenuate this decrease (Table 3).

A decrease in basal BS output was observed in LPS and OCA groups compared to the control group. In the co-treated group, BS output was even lower than in the LPS group.

As for total GSH output, a drop in this parameter was observed in the LPS group compared to the control group, and this decrease was partially counteracted by OCA coadministration.

### 3.3. Bsep and Mrp2 Transport Function

**Figure 1:**
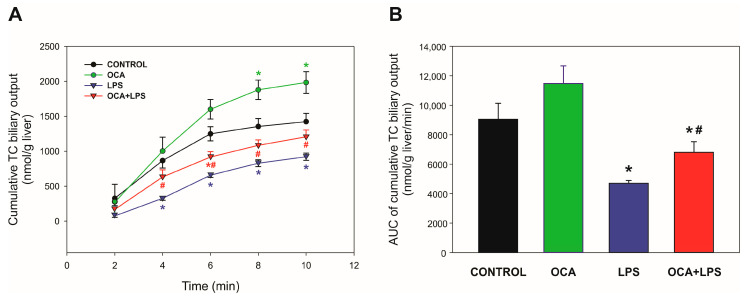
(**A**) Cumulative biliary excretion of taurocholate (TC). Bile samples were collected every 2 min for 10 min, after a single, i.v. dose of 8 μmol/100 g of b.w. of TC. (**B**) Area under the curve (AUC) of cumulative biliary excretion of TC. LPS, lipopolysaccharide; OCA, obeticholic acid. Data are shown as mean ± SEM. * *p* < 0.05 vs. control; ^#^ *p* < 0.05 vs. LPS. *n* = 4–5.

Bsep transport activity was evaluated through the excretion of its substrate, TC. This model BS was injected as a bolus, and then the cumulative biliary excretion of this compound was monitored. As can be seen in Figure 1, the cumulative biliary excretion of TC in the LPS-treated group was decreased compared to the control group. In contrast, a partial increase in TC excretion was observed in co-treated animals, suggesting an improved Bsep transport function. In the OCA-treated group, a tendency towards increased TC excretion was observed, which became significant at the end of the bile collection period, suggesting a stimulatory effect of OCA per se on Bsep transport function.

**Figure 2:**
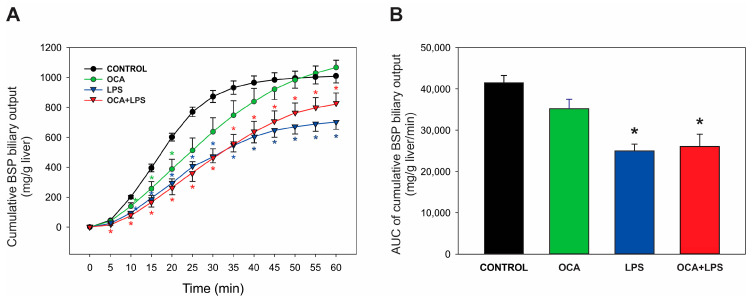
(**A**) Cumulative biliary excretion of bromosulfophthalein (BSP). Bile samples were collected every 5 min over a 60 min period, after the administration of a single, i.v. dose of BSP (3 mg/100 g b.w.). (**B**) Area under the curve (AUC) of cumulative biliary excretion of BSP. LPS, lipopolysaccharide; OCA, obeticholic acid. Data are shown as mean ± SEM. * *p* < 0.05 vs. control. *n* = 4–5.

Mrp2 activity was evaluated through the biliary excretion of the exogenous substrate, BSP. As shown in Figure 2, a decrease in the cumulative biliary excretion of BSP was observed in the LPS group, and this decrease was not counteracted by OCA pretreatment. OCA alone appeared to delay BSP excretion during the initial phases of bile collection; however, by the end of the experiment, cumulative BSP excretion was similar to that of the control group.

### 3.4. Canalicular and Basolateral Hepatocellular Transporter Gene Expression

A decrease in the mRNA levels of the canalicular transporters Bsep and Mrp2 was observed in LPS-treated animals compared to the control group (Table 4). Administration of OCA to LPS-treated rats partially prevented this reduction.

In addition, mRNA levels of some basolateral transporters of interest were evaluated. mRNA levels of Mrp3, the main basolateral extrusion BS transporter in the rat [42], were significantly increased by OCA and LPS when administered separately, and even more so when they were given in combination. On the other hand, no change was observed in mRNA levels of Mrp4, another putative basolateral BS export pump [43]. mRNA levels of the basolateral uptake transporters, Oatp2 and Ntcp, were also evaluated. For both transporters, a decrease in mRNA in the LPS group and an increase in the OCA group were observed compared to the control group. However, in the OCA + LPS group, only a minor increase of Ntcp but not of Oatp2 mRNA levels was observed.

### 3.5. Canalicular and Basolateral Transporter Protein Expression

**Figure 3:**
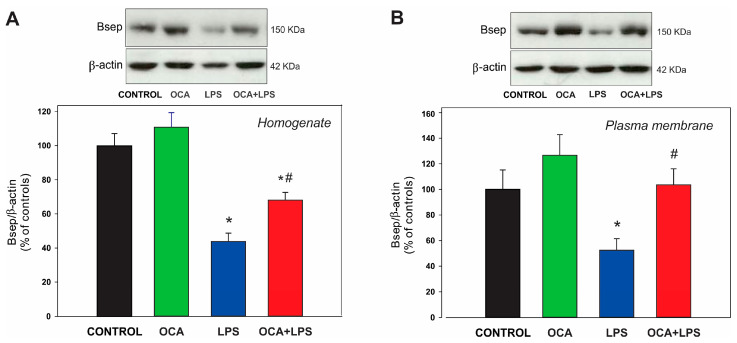
(**A**) Immunoblotting of Bsep in liver homogenates. (**B**) Immunoblotting of Bsep in canalicular plasma membrane. Densitometric quantification, normalized to β-actin, is presented as a percentage of the control group, which was set to 100%. LPS, lipopolysaccharide; OCA, obeticholic acid. Data are shown as mean ± SEM. * *p* < 0.05 vs. control; ^#^ *p* < 0.05 vs. LPS. *n* = 5.

Bsep protein expression was significantly reduced in the LPS-treated group, both at plasma membrane and total homogenate levels (Figure 3). Bsep content was only partially increased in total homogenate by OCA cotreatment, but fully normalized in plasma membrane fraction, suggesting that the latter increase was not only due to enhanced Bsep synthesis but also to normalized plasma membrane localization. A tendency toward higher Bsep levels in the plasma membrane, but not in the homogenate, was also observed in the OCA group, possibly reflecting an improvement in Bsep localization to the plasma membrane.

**Figure 4:**
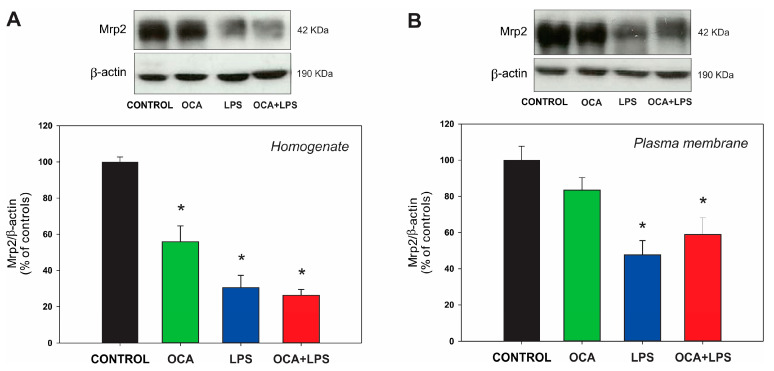
(**A**) Western blot analysis of Mrp2 in liver homogenate. (**B**) Western blot analysis of Mrp2 in liver plasma membranes. Densitometric quantification, normalized to β-actin, is presented as a percentage of the control group, which was set to 100%. LPS, lipopolysaccharide; OCA, obeticholic acid. Data are shown as mean ± SEM. * *p* < 0.05 vs. control. *n* = 5.

A decrease in Mrp2 protein expression levels was observed in total homogenate and in plasma membrane fraction in the LPS-treated group (Figure 4). In the co-treated group, no increase in protein level was observed in either the homogenate or the plasma membrane fraction, although a tendency toward increased Mrp2 levels was noted in the latter. OCA per se reduced Mrp2 levels in total homogenate, but this decrease was not reflected in the plasma membrane fraction. This suggests again an enhancement of Mrp2 plasma membrane localization induced by OCA per se, similar to what we found for Bsep.

**Figure 5:**
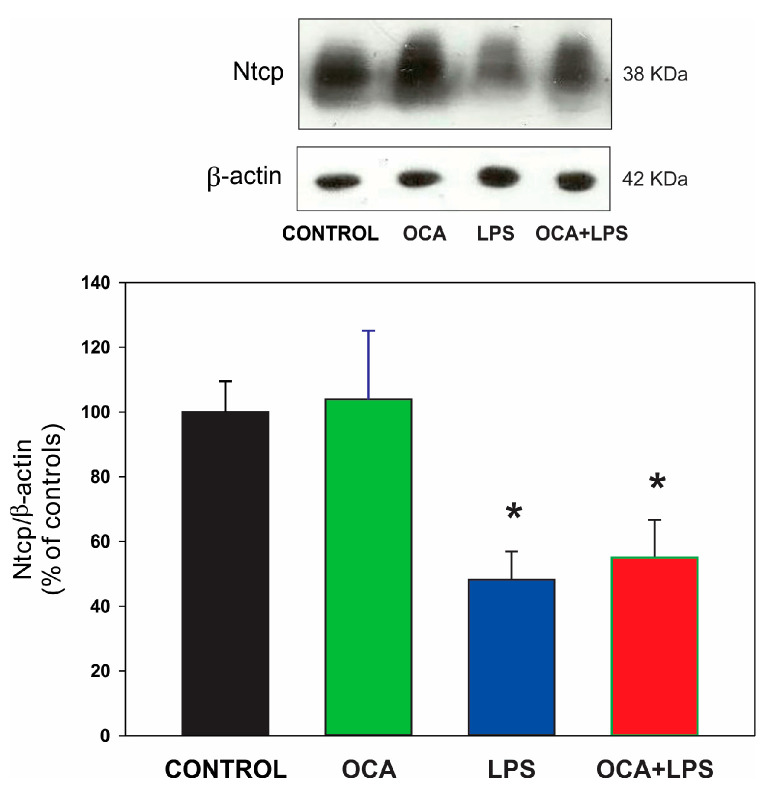
Protein expression of Nctp in liver homogenate by Western blot analysis. Densitometric quantification, normalized to β-actin, is presented as a percentage of the control group, which was set to 100%. LPS, lipopolysaccharide; OCA, obeticholic acid. Data are shown as mean ± SEM. * *p* < 0.05 vs. control. *n* = 4.

For Ntcp, a decreased protein expression in LPS-treated animals was observed, and this decrease was not counteracted by OCA pretreatment (Figure 5).

### 3.6. Bsep and Mrp2 Localization by Immunofluorescence Followed by Confocal Microscopy

**Figure 6:**
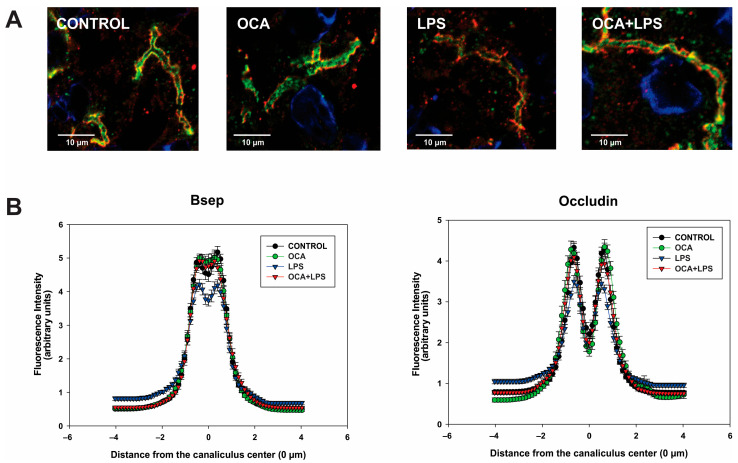
Analysis of Bsep localization by immunofluorescence and confocal microscopy. (**A**) Representative confocal images depicting dual immunostaining of Bsep (green) and occludin (red; marker delineating the bile canaliculus edge). Note that LPS-induced endocytic internalization of Bsep is partially attenuated by obeticholic acid (OCA). (**B**) Densitometric profiles quantifying Bsep- and occludin-associated fluorescence intensity for both along an 8-μm line drawn perpendicular to the bile canaliculus axis, spanning from −4 μm to +4 μm relative to the canalicular center, corresponding to the upper confocal images. Data represent mean ± SEM from analyses of 32 to 47 individual canaliculi.

Figure 6A shows representative images of Bsep localization in liver sections of control and treated animals. Bsep was stained in green and occludin in red, the latter used to mark the edge of the canaliculus.

In both control and OCA-treated animals, Bsep was predominantly localized at the canalicular membrane. However, when the animals were treated with LPS, a significant internalization of Bsep beyond the bile canaliculus boundaries was observed, as indicated by a decrease in the fluorescence intensity in the canalicular area together with increased fluorescence at a greater distance from the canaliculus (*p* < 0.001 vs. the control group). OCA co-administration effectively blocked the translocation of Bsep away from the canalicular membrane, preventing its accumulation in pericanalicular endosomes. The fluorescence intensity profiles associated with the transporter, recorded along a line perpendicular to the bile canaliculus, are presented in Figure 6B. Occludin profiles showed a conserved canalicular width, confirming that the observed changes in Bsep localization are not artifacts of alterations in canalicular structure.

**Figure 7:**
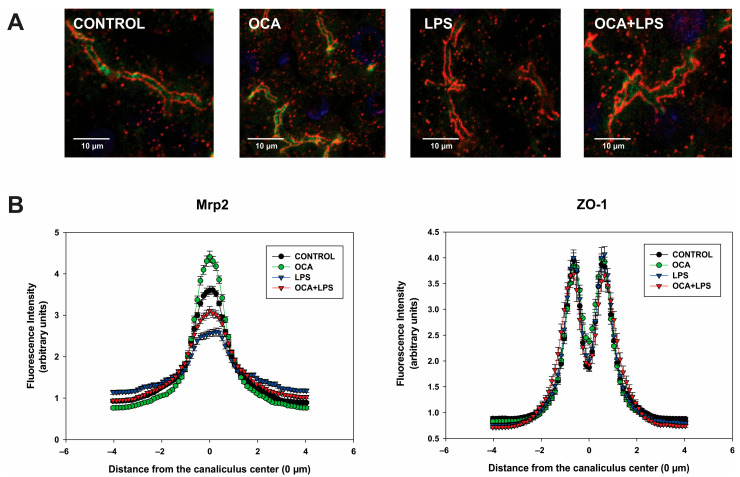
Analysis of Mrp2 localization by immunofluorescence and confocal microscopy. (**A**) Representative confocal images depicting dual immunostaining of Mrp2 (green) and ZO-1 (red; marker delineating the bile canaliculus boundary). Note that LPS-induced endocytic internalization of Mrp2 is partially attenuated by obeticholic acid (OCA). (**B**) Densitometric profiles quantifying Mrp2- and ZO-1-associated fluorescence intensity for both along an 8-μm line drawn perpendicular to the bile canaliculus axis, spanning from −4 μm to +4 μm relative to the canalicular center, corresponding to the upper confocal images. Data represent mean ± SEM from analyses of 32 to 47 individual canaliculi.

Similar results were observed for Mrp2 localization analysis, where ZO-1 instead of occludin was used to delimit the canaliculus edge (Figure 7). However, improvement of Mrp2 localization in the OCA + LPS group was partial in this case (*p* < 0.01 vs. both control and LPS groups).

## 4. Discussion

Extrahepatic bacterial infection and the subsequent host inflammation (sepsis) account for approximately 20% of cases of jaundice [44], and hyperbilirubinemia is a strong, independent risk factor for mortality [45]. The hyperbilirubinemia observed in this condition often reflects cholestasis, as it is associated with an increase in serum BS levels [46]. Therefore, cholestasis would be an important risk factor for mortality in sepsis, and pharmacological strategies to prevent or reverse this condition are urgently needed. In this study, we show that OCA has several transcriptional and post-transcriptional anticholestatic effects in the sepsis model of cholestasis induced by LPS in rats that may be relevant for clinical applications.

Serum ALP levels were fully normalized by OCA co-treatment (see Table 2). This parameter, in the cholestatic context, most likely reflects intracellular accumulation of BSs in hepatocytes throughout the cholestatic period; BSs both stimulate ALP synthesis and remove this enzyme from the plasma membrane, causing elevated serum levels [47]. Therefore, the normalization of ALP levels in the OCA + LPS group likely indicates that BSs have been more efficiently cleared from hepatocytes in this group. BS elimination under basal conditions does not seem to have been due to enhancement of Bsep-mediated BS biliary excretion (see Table 3), but rather to basolateral efflux of BSs due to the concomitant induction of Mrp3 (see Table 4), which redirects BSs towards sinusoidal blood for urinary excretion. Cholestasis is pathologically associated with the excessive accumulation of toxic BSs, which drive hepatocellular injury [48], and the ability of OCA to prevent this key pathogenic mechanism by upregulating Mrp3 expression underscores its anticholestatic properties. In addition, OCA improved Bsep-mediated canalicular excretion as well. However, the improvement of Bsep transport function becomes apparent only under BS overload conditions, as the model BS, TC, was more efficiently cleared via bile when the liver was overloaded with this BS (see Figure 1). It is likely that, under TC overloading conditions, unlike basal conditions, Mrp3 is closer to saturation due to its relatively low capability (i.e., low *V*max) to transport TC compared to Bsep [49]. Even when the relative contribution of Mrp3 to BS depuration may diminish due to this reason under TC overloading conditions, sinusoidal efflux still seems to compete with Bsep for TC transport, as a partial but not total recovery of TC biliary excretion was recorded despite full normalization of Bsep levels in its membrane domain in the OCA + LPS group (see Figure 3).

Interestingly, when administered alone, OCA also reduced ALP levels compared to control rats (see Table 2). This was likely due to the upregulation that OCA exerts, per se, on Mrp3 expression (see Table 4), in line with previous results [50], thus leading to reduced intracellular BS accumulation. This, in turn, attenuates the upregulation and membrane removal of ALP, ultimately resulting in lower serum ALP levels. Furthermore, the redirection of BSs toward the Mrp3-mediated export pathway reduces the availability of substrate for the Bsep-mediated biliary route. This shift leads to a marked decrease in biliary BS output and the associated reduction in BF observed in the OCA-alone group, as also seen in the OCA + LPS group (see Table 2). This illustrates the concept that a reduction in BF does not necessarily indicate cholestatic failure, as it was not associated with the accumulation of toxic BSs in either case, as evidenced by the low serum ALP levels.

Bsep mRNA expression and protein levels, both in total homogenate and the plasma membrane, were also evaluated (see Table 4 and Figure 3, respectively). Although *BSEP* is a well-known target gene of FXR in humans [51,52], Bsep expression at both the mRNA and protein levels was not significantly increased when OCA was administered alone in our whole rat model (see Table 4 and Figure 3), in agreement with previous reports in rats [24,53] and mice [25]. This finding is in line with data from a comparative transcriptomic analysis of the effect of OCA on FXR target genes in human precision-cut liver slices and in mouse liver, which showed that BSEP/Bsep was strongly upregulated by OCA only in human tissue [54].

Unlike the lack of effect of OCA per se on Bsep expression, this compound significantly increased Bsep expression in LPS-treated rats at both the mRNA and protein levels (see Figure 3 and Table 4). Transcriptional downregulation of Bsep in LPS-induced cholestasis is mainly due to cytokines released during the acute-phase response [55], and cytokine-mediated repression of FXR has been suggested to be a crucial causal factor [56]. OCA may have improved Bsep expression in LPS-treated rats by several mechanisms, which could act in concert: (i) as shown here (see Table 1) OCA partially prevented the elevation of the proinflammatory cytokine IL-1β, which has been selectively involved in the Bsep downregulation induced by LPS [55]; (ii) Bsep downregulation in LPS-treated rats is expected to be further aggravated by the inflammatory state induced by accumulated BSs [57], and the enhanced BS depuration induced by OCA may have helped attenuate this alteration; (iii) OCA may have stimulated the remaining Bsep transactivating activity of FXR in LPS-treated rats, even though it failed to do so under control, non-inflammatory conditions. For example, FXR transactivating activity is normally increased post-translationally via SUMOylation [58], but this stimulatory epigenetic modification is rapidly impaired upon LPS treatment, and OCA has been shown to efficiently inhibit this deleterious effect of LPS on FXR transactivation [59]. Partial, but not total, counteracting effects of OCA on Bsep expression may reflect partial FXR-mediated transactivation due to the limited capability of FXR to activate its target genes under inflammatory conditions. It is worth noting that, in addition to downregulating FXR expression, cytokines inhibit the DNA binding of FXR to the Bsep IR-1 response element [59,60]. Additionally, FXR binds to DNA as a heterodimer with RXRα to transactivate target genes, but RXRα levels are reduced in LPS-induced cholestasis [56,61]. However, RXRα can be partially replaced by other RXR isoforms that are not affected by cytokines, such as RXRβ and RXRγ [56]. Interestingly, binding of FXR to RXR is impaired by proinflammatory cytokines via a c-jun-N-terminal kinase-dependent RXRα phosphorylation, but FXR transactivating ligands suppress this effect [62].

Similarly to Bsep, Mrp2 mRNA levels were increased only in the co-treated OCA + LPS group (see Table 4), in line with previous studies in rats [24,50] and in cultured human hepatocytes [63]. A major role in this protective effect of OCA is likely due to its ability to fully counteract the release of TNF-α induced by LPS (see Table 2), as this cytokine selectively mediates the downregulation of Mrp2 by LPS [55]. However, unlike Bsep, the upregulation of Mrp2 at the mRNA level was not reflected at the protein level, either in the homogenate or in the plasma membrane (Figure 4). Differences from Bsep in post-translational events, such as protein modifications that differentially affect Mrp2 degradation, or changes in sorting and targeting to its membrane domain, may be involved. For example, unlike Bsep, which undergoes proteasomal degradation [64], Mrp2 is trafficked to lysosomes after endocytosis [65], and lysosomal protein degradation is exacerbated in endotoxemic rats [66]. Coincidentally, we previously found a similar lack of improvement in Mrp2 protein levels after ursodeoxycholic acid administration in a similar model of LPS-induced cholestasis [35]. Mrp2 transport activity, assessed by measuring the biliary excretion of the exogenous Mrp2 substrate BSP, was also not modified either, as expected from the lack of increase in Mrp2 protein expression (see Figure 2). A tendency toward diminished BSP excretion when OCA was administered alone was nevertheless apparent, which may reflect the basolateral efflux of the glutathione-conjugated BSP metabolite via Mrp3, as Mrp3 efficiently transports glutathione conjugates [67]. Since Mrp2 is the main transporter of GSH into bile [68], the lack of improvement in Mrp2 transport function in the co-treated OCA + LPS group rules out the possibility that the increased GSH biliary excretion observed in this group (see Table 3) is due to enhanced Mrp2 activity. GSH biliary excretion is also critically dependent on the hepatocellular redox status, as oxidized GSH is rapidly exported into bile following an oxidative challenge, leading to intracellular GSH depletion [69] and, consequently, impaired excretion. It is, therefore, likely that OCA improves the redox status of endotoxemic rats by counteracting the two main sources of pro-oxidant damage: proinflammatory cytokines [70] and the accumulation of intracellular BSs associated with FXR downregulation [71]. Further studies on the preventive effects of OCA on the hepatic redox status in LPS-treated rats are required to confirm this hypothesis.

The basolateral BS uptake transporters, Ntcp and Oatp2, were also evaluated. In agreement with previous reports [5,14,72,73], both transporters were downregulated by LPS via transcriptional mechanisms (see Figure 5 and Table 4). These downregulations, together with the upregulation of basolateral BS efflux via Mrp3, are considered adaptive rather than deleterious responses, as they help prevent BSs from accumulating inside hepatocytes [74]. The downregulation of BS uptake transporter occurs, in part, through the BS-activated, FXR-mediated upregulation of the transcriptional repressor, Shp [75], and, therefore, accumulated BSs may have been a triggering factor. Although intracellular BS levels were normalized by OCA co-treatment, and this BS-mediated negative feedback should, thus, have been abolished, it is likely that direct effects of OCA on FXR transactivation of the Shp promoter were sufficient to sustain these beneficial downregulations in the OCA + LPS group.

A relevant finding of this study is that OCA significantly improved Bsep localization to its membrane domain in endotoxemic rats, as indicated by the full normalization of Bsep plasma membrane levels despite a decrease in total (intracellular plus plasma membrane) Bsep content (see Figure 3). These results were confirmed by immunostaining studies followed by confocal microscopy and image analysis (see Figure 6). The release of proinflammatory cytokines induced by LPS is thought to be a key factor in Bsep internalization [76,77], as the counteraction of cytokine release by dexamethasone has been shown to prevent Mrp2 internalization [14], and Mrp2 shares with Bsep common mechanisms of endocytic internalization [78]. However, the mitigation of the inflammatory state by OCA was only partial, with IL-1β levels remaining significantly elevated in the OCA + LPS group. Therefore, post-transcriptional effects of OCA not mediated by its anti-inflammatory action should also be considered. OCA has demonstrated rapid protective effects against acute cholestasis induced by lithocholic acid in rats, which cannot be attributed to transcriptional changes but rather to post-transcriptional mechanisms. Since we have previously shown that lithocholic acid–induced cholestasis involves Bsep internalization as a major pathomechanism [79], it is likely that OCA protects against LPS-induced Bsep internalization via short-term activation of anticholestatic transduction pathways. Although the specific mediators of this OCA effect are currently unknown, other BSs are known to activate signaling pathways that protect against transporter endocytic internalization. For example, the anticholestatic BS, tauroursodeoxycholate, induces dual activation of p^38^ MAPK and ERK1/2 MAPKs [80,81], which promote rapid targeting and insertion of intracellular canalicular transporters. Interestingly, both signaling molecules can also be activated by other BSs, including the OCA parent compound, chenodeoxycholic acid [82]. Studies aimed at elucidating this novel, post-transcriptional anticholestatic mechanism of OCA and the signaling pathways involved are warranted. This issue is particularly relevant because, unlike in rodents where changes in hepatocellular transporter expression during cholestasis are mostly transcriptional, in human cholestasis, these alterations are often post-transcriptional in nature [9]. This is especially true for human endotoxemia-induced cholestasis, where LPS downregulates both BSEP and MRP2 exclusively via post-transcriptional mechanisms [14]. This downregulation likely involves carrier endocytosis followed by degradation, as endocytosed transporters exposed to sustained LPS stimulation are thought to be targeted to degradation compartments [83].

Although our results are directly relevant to sepsis-induced cholestasis, clinically significant endotoxemia also occurs under non-septic conditions, where hepatocellular transporters may likewise become dysfunctional [84]. For example, patients with alcoholic liver disease [85], metabolic-associated fatty liver disease [86], or parenteral nutrition-associated cholestasis [87] may have portal vein and systemic endotoxemia. In addition, elevated levels of proinflammatory cytokines, which mediate the effects described here for LPS, have been reported in a wide range of other inflammatory types of cholestasis [2,10].

## 5. Conclusions

OCA exerts multiple transcriptional and post-transcriptional anticholestatic mechanisms in LPS-induced cholestasis, which act in concert to regulate hepatocellular transporters involved in bile formation and BS homeostasis, otherwise dysregulated in this inflammatory context. These mechanisms include enhanced biliary BS clearance through improved Bsep expression and localization, accelerated basolateral BS efflux via Mrp3, and reduced BS uptake through downregulation of Ntcp and Oatp2. Collectively, these effects are expected to reduce intrahepatic BS accumulation characteristic of cholestasis, thereby attenuating cholestatic injury. If these results are confirmed in clinical settings, cholestatic patients with endotoxemia, and potentially those with other inflammatory cholestatic diseases, may benefit from the well-established anti-inflammatory effects of OCA, its FXR-mediated regulation of hepatocellular transporter expression, and possibly from signaling mechanisms that ensure appropriate localization of newly synthesized transporters, thereby preventing excessive degradation.

## Figures and Tables

**Table 1:** Sequence of the primers used in real-time PCR.

Gen Target	Primer	Primer Sequence (5′ ⟶ 3′)
*Bsep*	Forward	CCGAAGGCTCAGGGTATTGG
	Reverse	ATCAGGTGACATGGTGGCAG
*Mrp2*	Forward	ACCTTCCACGTAGTGATCCT
	Reverse	ACCTGCTAAGATGGACGGTC
*Mrp3*	Forward	GTGCTGAAGAATTTGACTCTG
	Reverse	GACCAGGACCCGGTTGTAGTC
*Mrp4*	Forward	CTGCGGTCACAGTCCTCTTT
	Reverse	GTGCAGAGTCTGGGAAGCAT
*Ntcp*	Forward	TCCTTCCCCTAATGGCCTGA
	Reverse	CGTCGACGTTCGTTCCTTTT
*Oatp2*	Forward	TCCTCTGCATGTGCAAAACTTC
	Reverse	TGGCACACTCTGAAGAGTCTAAT
18s rRNA	Forward	GTAACCCGTTGAACCCCATT
	Reverse	CCATCCAATCGGTAGTAGCG

**Table 2:** Plasma levels of ALP and the proinflammatory cytokines, IL-1β and TNF-α.

	Control	OCA	LPS	OCA + LPS
ALP (U/L)	170 ± 8	146 ± 7 *	368 ± 52 *	183 ± 18 ^#^
IL-1β (pg/mL)	26 ± 24	36 ± 22	860 ± 153 *	266 ± 56 *^#^
TNF-α (pg/mL)	21 ± 3	41 ± 8	52 ± 8 *	13 ± 3 ^#^

ALP, alkaline phosphatase; IL-1β; interleukin-1β; LPS, lipopolysaccharide; OCA; obeticholic acid; TNF-α, tumor necrosis factor-α. * *p* < 0.05 vs. control; ^#^ *p* < 0.05 vs. LPS; *n* = 4–5.

**Table 3:** BF and biliary excretion of total BSs and total GSH.

Biliary Parameters	Control	OCA	LPS	OCA + LPS
Bile flow (μL/min per g liver)	3.3 ± 0.2	3.2 ± 0.1	2.3 ± 0.1 *	2.5 ± 0.1 *
Total BS output (nmol/min per g liver)	45 ± 6	13 ± 2 *	25 ± 3 *	7 ± 1 *^#^
Total GSH output (nmol/min per g liver)	1534 ± 171	1682 ± 230	578 ± 47 *	787 ± 50 *^#^

BF, bile flow; BS, bile salt; GSH, glutathione; LPS, lipopolysaccharide; OCA; obeticholic acid. * *p* < 0.05 vs. control; ^#^ *p* < 0.05 vs. LPS; *n* = 4–9.

**Table 4:** mRNA expression of hepatocellular transporters by real-time PCR.

mRNA Levels (% of Mean Control Values)	Control	OCA	LPS	OCA + LPS
Bsep	100 ± 7	112 ± 13	40 ± 9 *	79 ± 19 ^#^
Mrp2	100 ± 14	132 ± 9	47 ± 4 *	71 ± 5 ^#^
Mrp3	100 ± 10	215 ± 54 *	179 ± 60 *	289 ± 29 *^#^
Mrp4	100 ± 12	101 ± 4	100 ± 11	105 ± 12
Oatp2	100 ± 11	151 ± 17 *	5 ± 1 *	4 ± 1 *
Ntcp	100 ± 3	158 ± 20 *	45 ± 2 *	68 ± 7 *^#^

LPS, lipopolysaccharide; OCA; obeticholic acid. * *p* < 0.05 vs. control; ^#^ *p* < 0.05 vs. LPS; *n* = 7–9.

## Data Availability

The data presented in this study are available on request from the corresponding authors.

## Abbreviations

The following abbreviations are used in this manuscript:

ALP	Alkaline phosphatase
BF	Bile flow
BS	Bile salt
Bsep	Bile salt export pump
BSP	Bromosulfophthalein
FXR	Farsenoid X receptor
GSH	Glutathione
LPS	Lipopolysaccharides
Mrp2	Multidrug resistance-associated protein 2
Mrp3	Multidrug resistance-associated protein 3
Ntcp	Na^+^/taurocholate cotransporting polypeptide
Oatp2,	Organic anion-transporting polypeptide
RXRα,	9-cis-retinoic acid receptor-α
TC	Taurocholate
